# The economic burden of diabetes to French national health insurance: a new cost-of-illness method based on a combined medicalized and incremental approach

**DOI:** 10.1007/s10198-017-0873-y

**Published:** 2017-02-11

**Authors:** Grégoire de Lagasnerie, Anne-Sophie Aguadé, Pierre Denis, Anne Fagot-Campagna, Christelle Gastaldi-Menager

**Affiliations:** Strategy and Research Department, National Health Insurance (CNAMTS), 50 Avenue du Pr André Lemierre, 75986 Paris Cedex 20, France

**Keywords:** Diabetes, Cost of illness, Econometrics, Health administrative databases, I180, H510, C180, C550

## Abstract

A better understanding of the economic burden of diabetes constitutes a major public health challenge in order to design new ways to curb diabetes health care expenditure. The aim of this study was to develop a new cost-of-illness method in order to assess the specific and nonspecific costs of diabetes from a public payer perspective. Using medical and administrative data from the major French national health insurance system covering about 59 million individuals in 2012, we identified people with diabetes and then estimated the economic burden of diabetes. Various methods were used: (a) global cost of patients with diabetes, (b) cost of treatment directly related to diabetes (i.e., ‘medicalized approach’), (c) incremental regression-based approach, (d) incremental matched-control approach, and (e) a novel combination of the ‘medicalized approach’ and the ‘incremental matched-control’ approach. We identified 3 million individuals with diabetes (5% of the population). The total expenditure of this population amounted to €19 billion, representing 15% of total expenditure reimbursed to the entire population. Of the total expenditure, €10 billion (52%) was considered to be attributable to diabetes care: €2.3 billion (23% of €10 billion) was directly attributable, and €7.7 billion was attributable to additional reimbursed expenditure indirectly related to diabetes (77%). Inpatient care represented the major part of the expenditure attributable to diabetes care (22%) together with drugs (20%) and medical auxiliaries (15%). Antidiabetic drugs represented an expenditure of about €1.1 billion, accounting for 49% of all diabetes-specific expenditure. This study shows the economic impact of the assumption concerning definition of costs on evaluation of the economic burden of diabetes. The proposed new cost-of-illness method provides specific insight for policy-makers to enhance diabetes management and assess the opportunity costs of diabetes complications’ management programs.

**Electronic supplementary material:**

The online version of this article (doi:10.1007/s10198-017-0873-y) contains supplementary material, which is available to authorized users.

## Introduction

A better understanding of the economic burden of diabetes constitutes a major public health challenge for health insurers in order to identify ways to improve diabetes follow-up and control the dynamics of diabetes-related expenditure [1–3]. In France, diabetes is a major public health problem, as about 3 million patients who received care for diabetes in 2012, i.e., 4.6% of the whole population [1]. In view of the growing prevalence of the main risk factors for diabetes (ageing of the population, obesity, and sedentary lifestyle), as well as the growing population, this situation is likely to worsen with time [1, 4–7]. The severity of diabetic complications, such as cardiovascular disease, renal failure, and amputations [2], and the association between diabetes and excess risk of other chronic diseases such as certain cancers [3], justify active management of this disease [1].

The scope of costs included to evaluate the economic burden of a disease is the subject of intense discussion in the literature [8–13]. When the definition of costs is restricted to health care expenditure, excluding costs related to impaired quality of life, there is still a persistent debate between supporters of a comprehensive expenditure approach and supporters of a more restrictive approach, targeted to specific expenditure related to management of the disease. Between these two extremes, an intermediate, so-called incremental, definition has also been widely used [12, 13]. This method consists of measuring the excess expenditure related to the disease by comparing the expenditure of individuals with the disease to that of individuals without the disease but presenting similar demographic and socioeconomic characteristics in order to isolate the costs specifically due to the disease.

The three most commonly used expenditure-based approaches [8] address different and complementary economic and epidemiological questions. First, the global comprehensive approach provides an overall picture of all expenditure of a population with a particular disease (type of care, concentration, dispersion), whether or not this expenditure is related to the disease [6]. Second, medicalized approaches can be used to distinguish expenditure that is highly specific to the disease from other types of expenditure, with an a priori definition of specific expenditure. These approaches provide insight into the costs of the various types of care used to treat the disease. Third, incremental approaches can be used to distinguish overconsumption of a particular population due to the illness, its complications and the impaired health status related to the disease. These methods can be used to estimate the overall costs of the disease without identifying, ex-ante, the expenditure specifically related to the disease.

The present study was designed to contribute to the international literature by comparing the various approaches recently used in cost-of-illness studies to evaluate the financial burden of diabetes [14]. It also presents a new approach based on a combination of existing methods to distinguish direct costs specifically due to diabetes by means of a medicalized approach from costs related to complications and impaired health status by means of an incremental approach. In particular, we identified reimbursements specific to antidiabetic treatments, as well as reimbursements related to the main complications of diabetes (cardiovascular diseases and chronic renal failure). Reimbursements of diabetes-related health care expenditure were extracted from the Système National d’Information InterRégimes de l’Assurance Maladie [National Health Insurance Information System] (SNIIRAM) database in 2012 [15].

In the following section, we will describe the data used for this study. We will then describe the methodology used to estimate national health insurance reimbursements effectively related to diabetes. Finally, the results of the study will be presented, followed by a discussion.

## Data

The Système National d’Information InterRégimes de l’Assurance Maladie [National Health Insurance Information System] (SNIIRAM), designed to provide a better understanding and more accurate evaluation of quality of care, health care use and associated expenditure, was set up in France in the early 2000s [15]. While some administrative databases in other countries are only representative of a subpopulation (e.g., in the U.S., Medicare data are representative of individuals 65 years and older), the SNIIRAM database contains data on all reimbursed health care expenditure (inpatient, outpatient, and cash payments) for the entire population living in France. It also includes sociodemographic, medical, and administrative data concerning these beneficiaries (age, gender, diagnoses of long-term diseases eligible for 100% reimbursement, diagnoses reported during hospitalizations, town of residence, date of death) [15]. The SNIIRAM database is therefore probably one of the largest national health databases in the world, in contrast to databases in northern European countries, in which data are representative of the entire population, but based on a smaller number of observations.

Reimbursements of diabetes-related health care expenditure were extracted from the SNIIRAM database in 2012 for people insured by the French health insurance general scheme and local schemes (86% of the French population, 59 million individuals), with the approval of the French data protection authority (Commission Nationale Informatique et Liberté). The French health insurance general scheme covers salaried workers, retired private sector individuals, and, more generally, all individuals not covered by a specific scheme (farmers, self-employed, special schemes), and their relatives (76% of the population living in France). Local schemes provide health insurance coverage for civil servants, students, and hospital staff (10% of the population living in France).

The scope of expenditure considered in this study included outpatient care (office visits, drugs, medical devices, nursing care, laboratory tests), and hospital expenditure, including public and private medical, surgical and obstetric (MSO) hospital stays, aftercare and rehabilitation (CR) and psychiatric admissions. Cash payments, such as daily allowances or disability pensions were also taken into account, but only for those paid by the general scheme, as data from local schemes may be incomplete or missing. The expenditure studied in this paper represented a total of €124 billion in 2012 that can be linked to general health scheme and local scheme beneficiaries.

## Method

### Identification and characteristics of the diabetic population in 2012

An algorithm was used to qualify a patient as diabetic if and only if this patient had received at least three reimbursements for antidiabetic drugs (oral or insulin) in 2012 (at least two reimbursements if at least one large pack size was dispensed), or in 2011 in order to avoid censorship effects, or when this patient had been allocated long-term disease (LTD) status for diabetes in 2012. The list of antidiabetic drugs corresponds to class A10 of the Anatomical Therapeutic Chemical (ATC) classification, with the exception of benfluorex [16]. In addition to age and gender, two variables were used as a proxy to characterize the individual’s financial situation: complementary universal health insurance coverage (CMU-C) and an ecological deprivation index [17, 18]. Complementary universal health insurance coverage (“couverture maladie universelle complémentaire” or CMU-C) is provided by national health insurance schemes to people with incomes lower than a defined ceiling (€7934 for a single person as of July 2012). The deprivation index reflects a major part of spatial socioeconomic heterogeneity based on four indicators (median household income, percentage of high school graduates in the population aged 15 years and older, percentage of blue-collar workers in the active population, and the unemployment rate) homogeneously throughout metropolitan France. This index is routinely used to observe, analyze, and manage spatial health inequalities.

### Definitions of the scope of diabetes-related reimbursed expenditure

Three main methods that have been recently used to estimate the financial burden of a disease [8] were used in this study (Table 1). In addition, for the first time in the literature to the best of our knowledge, a combination of two of these methods was used in order to propose a new approach (Method 4).

#### Method 1: global comprehensive definition

The global comprehensive definition was initially adopted in order to establish an estimate of all expenditure (for diabetes or for any other disease) reimbursed to patients with diabetes and to therefore characterize the burden of reimbursements paid to these patients compared to all reimbursements to all patients [8]. The sum of all expenditure reimbursed to patients with diabetes was calculated. This global comprehensive analysis also allows a description of types of care used and the distribution of annual expenditure reimbursed to patients (mean, dispersion, concentration of expenditure).

#### Method 2: medicalized approach

In the context of the medicalized approach, reimbursements paid to the diabetic population for diabetes-specific expenditure were entirely and directly attributed to diabetes. The following types of outpatient expenditure were considered to be diabetes-specific: endocrinologist visits, reimbursements of medical devices on the “Liste des Produits et des Prestations remboursables” (LPP) [List of reimbursed medical devices and services] intrinsically related to diabetes (dip-sticks, insulin pens, and insulin pump materials), reimbursements of antidiabetic drugs (oral and insulin), reimbursements of blood glucose, and glycated hemoglobin (HbA1c) assays and reimbursements of podiatrist fees (fee set up by national health insurance to improve the prevention of diabetic foot lesions for patients at high risk). For inpatient care, reimbursements related to Medicine, Surgery, and Obstetrics (MSO) hospital stays for diabetes (as a principal or related diagnosis, corresponding to codes E10-E14 of the International Classification of Diseases, Tenth edition) were considered to be diabetes-specific and were also entirely attributed to diabetes-related reimbursements. The expenditure observed for patients not identified as having diabetes according to the algorithm, but who were admitted to hospital with a diagnosis of diabetes in 2012 (as a principal or related diagnosis) or who had received at least one reimbursement of podiatrist fees for diabetes in 2012 was also added to the diabetes-specific expenditure (to compensate for incomplete detection by the algorithm of a small number of patients with diabetes).

#### Method 3: incremental approach

The incremental approach includes both a regression-based approach and a matched-control approach, in which a control group of patients without the disease is used to estimate the cost of illness.

#### Method 3.1: regression-based incremental approach

The regression-based incremental approach is also commonly used in the literature [10, 12, 19]. A large number of papers have been published on modeling of health care expenditure in order to take into account two important characteristics of the distribution of health care expenditure: the large number of subjects with zero expenditure and the highly-skewed distribution (for a formal description of the various challenges involved in health care expenditure estimation models refer to [20–22]). The various models reported in the literature comprise two equations designed to take zero expenditure into account. The first equation models the individual’s decision to access health care services, i.e., the probability of having health care expenditure different from zero. The second equation determines the level of health care consumption in the subsample of individuals with health care expenditure different from zero.

These two equations can be estimated according to two models depending on the economic hypothesis adopted to characterize the relationship between the decision to access health care and the level of health care consumption. The Sample Selection Model is based on the hypothesis of a correlation between the two decisions. The second type of model is the Two-Part Model. This model is based on the hypothesis that the decision to access health care and the level of health care consumption are not correlated and that these two equations are independent. The Two-Part Model cannot conclude on a causal inference between exogenous variables and the level of health care expenditure because this model does not take into account individual heterogeneity, which certainly influences the probability of health care consumption and the level of health care consumption. However, the Two-Part Model is sufficient for prediction of health care expenditure, as this calculation does not analyze the effect of a particular variable [22].

The objective of the present study was to simulate the mean level of health care expenditure of the population rather than interpret and analyze coefficients of health care demand. Consequently, we adopted the hypothesis that there is no relationship between the decision to access health care and the level of health care consumption. We therefore exclusively estimated the second part of a Two-Part Model concerning only those people with at least one reimbursement detected in the SNIIRAM database. The level of health care consumption was estimated by the generalized linear model (GLM). We chose the most appropriate link function for our data log-link with a gamma distribution and tested the goodness of fit of this model [10] (see goodness of fit test results in Appendix 1).

The vector of control variables is composed of age, gender, and diabetes status. In order to calculate the annual spending attributable to diabetes, annual spending was initially predicted by using the coefficients of the GLM estimation using a GLM specification where *D*_*i*_ is healthcare spending and *X*_1,*i*_ are the explanatory variables used in the estimation. Health care consumption is predicted by:

$$\hat{E}(D_{i} |X_{1,i} ) = \exp (X^{\prime}_{1,i} \hat{\beta) }.$$

The hypothetical health care expenditure of patients with diabetes if they did not have diabetes was then estimated by applying a coefficient of 0 associated with diabetes in the health care expenditure equation. Diabetes-specific expenditure was estimated by the mean difference between these two predictions [10].

#### Method 3.2: matched-control incremental approach

A matched-control incremental approach was then performed for all spending to determine the impact of diabetes on health care expenditure [23, 24]. According to this method, the excess reimbursements attributable to diabetes were measured by determining the differential between reimbursements paid to patients with diabetes and those without diabetes. To calculate this excess reimbursement, we defined a control group of patients without diabetes stratified by 10-year age groups. The excess reimbursements related to diabetes were therefore estimated for each age-group as the difference between the expenditure of the diabetes population (case) and the expenditure of the population without diabetes (control). In other words, the reimbursed expenditure differential was estimated by gender and by 10-year age groups. Ten-year age groups were used rather than exact age groups in order to allow regional analysis of diabetes expenditure by means of the same methodology with a sufficient number of individuals in each group to provide significant and robust results. As the incremental approach is designed to identify costs that are causally related to diabetes (such as the costs related to complications of diabetes), no adjustment can be performed for variables causally related to diabetes.

#### Method 4: combination of medicalized and incremental approaches

Lastly, the global medicalized and matched-control incremental definitions were used in combination (Table 1) to distinguish health care expenditure specific to the management of diabetes (using the global medicalized approach) from that related to management of complications and/or excess health care consumption induced by impaired health status due to diabetes (using the incremental approach). Both of these methods have been used previously [10], but not necessarily in the same study in order to provide a better understanding of the expenditure attributable to diabetes. Firstly, diabetes-specific expenditure was entirely and directly attributed to diabetes according to the medicalized approach. Secondly, the matched-control incremental approach was then performed on the overall population to determine the impact of diabetes on the rest of health care expenditure (not specific to diabetes), as diabetes is a risk factor for certain chronic diseases. Excess reimbursements for diabetes-related complications, matched for age and gender, represent the cost of developing a specific disease for a patient with diabetes. The implicit hypothesis is that if diabetes complications could be eradicated, excess reimbursements would be zero. However, this simplifying assumption is not fully met, as factors other than age and gender may also be involved in the comparison between the health care expenditure of patients with or without diabetes [16, 25].

**Table 1:** Allocation of diabetes-related reimbursements

Method	Scope of health insurance reimbursements	Results
Method 1	Reimbursements in the population with diabetes	See “Global comprehensive approach: reimbursements paid to patients with diabetes”
Method 2	Medicalized approach: reimbursements specific to diabetes • Endocrinologist visits, dip-sticks, insulin pens and insulin pump materials, reimbursements of antidiabetic drugs (oral and insulin), reimbursements of blood glucose and glycated hemoglobin, Medicine, Surgery, and Obstetrics (MSO) hospital stays for diabetes	See “A global medicalized and incremental definition: diabetes-related reimbursed expenditure in France”
Method 3.1	Incremental definition: Regression-based approach • Estimation of the determinants of the level of health care expenditure as a function of diabetes by controlling for individual characteristics.	See “Regression-based incremental approach: spending attributable to diabetes”
Method 3.2	Incremental definition: Matched-control approach • Comparing all medical expenditure of patients with and patients without diabetes by gender and by 10-year age-groups	See “Incremental definition: matched-control approach”
Method 4	Combination of medicalized and matched-control incremental approaches	See “A global medicalized and incremental definition: diabetes-related reimbursed expenditure in France”

## Results

### Characteristics of the diabetic population in 2012

According to the algorithm used in this study, 2.9 million people with diabetes were identified among the 59 million general health scheme and local scheme beneficiaries in 2012. The main characteristics of patients with diabetes identified by this algorithm are described in Table 2. As expected, these patients were older than the general population with a mean age of 66 years versus 39 years, as the prevalence of diabetes increases very markedly with age. Diabetes also appears to be related to socioeconomic markers, as an over-representation of people with diabetes was observed in territories with lower socioeconomic status. One quarter of patients with diabetes in 2012 lived in territories with the lowest socioeconomic quintile (versus 20% for the general population) and only 16% lived in territories with the highest socioeconomic quintile.

**Table 2:** General descriptive statistics of the SNIIRAM database Source: CNAMTS\SNIIRAM

	Study population: General Health Scheme and Local schemes	Patients with diabetes (type 1 or 2)
Number of patients	59 million	2.9 million
Proportion of women	54%	48%
Age		
Mean age	39 years	66 years
Median age	38 years	66 years
Expenditure		
Total reimbursed expenditure	€124 billion	€19 billion
Mean reimbursement per individual	€2199	€6714
Ecological deprivation index		
Q1 (people living in territories with the highest socioeconomic index)	20%	16%
Q2	20%	18%
Q3	20%	19%
Q4	20%	21%
Q5 (people living in territories with the lowest socioeconomic index)	20%	25%
Complementary universal health insurance coverage for the less well off (CMU-C)		
% CMU-C (≤60 years)	11%	14%

### Global comprehensive approach: reimbursements paid to patients with diabetes

The sum of all reimbursements (health care consumption, daily allowances and disability pensions) for patients with diabetes, whether or not the expenditure was related to diabetes, was €19 billion, i.e., 15% of all general health scheme and local scheme reimbursements (€124 billion).

**Fig. 1:**
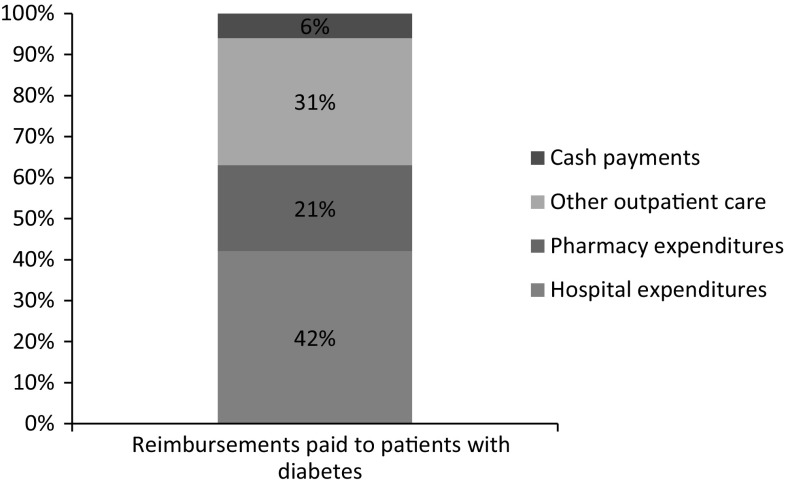
Breakdown of reimbursements to patients with diabetes Source: CNAMTS\SNIIRAM 2012

In 2012, patients with diabetes (mean age: 66 years) therefore received an average of €6714 of health insurance reimbursements. Hospital expenditure represented 42% of all reimbursements, pharmacy expenditure represented 21% and other outpatient care (medical fees, nursing care etc.) represented 31%, and cash payments (daily allowances and disability pensions) represented 6% of all reimbursements (Fig. 1).

A U-shaped relationship was observed between mean reimbursed expenditure and age, which is likely to be related to insulin therapy for people with type 1 diabetes at a younger age due to progression of the disease over time and the development of complications, as well as other diseases. Patients with diabetes under the age of 16 years received a mean reimbursement of €7000 (Table 3) versus €5500 for patients between the ages of 16 and 45 years and €6000 for patients between the ages of 46 and 65 years. In 2012, people with diabetes 65 years and older received a mean reimbursed expenditure of €7300. The mean reimbursement of insulin-treated patients was €12,200 versus €5200 for other people with diabetes. Finally, mean reimbursed expenditure for patients living in areas with the lowest socioeconomic index was €6845 versus €6469 for those living in territories with the highest socioeconomic index. This difference cannot be explained by differences in mean age, which was equal to 66 years in these two types of territories.

**Table 3:** Mean reimbursements to patients with diabetes in 2012 Source: CNAMTS\SNIIRAM 2012

	Mean reimbursement paid by general health scheme and local schemes
Age	
Less than 16 years	€6986
16–45 years	€5514
46–64 years	€6015
65 years and older	€7324
Ecological deprivation index	
Q1 (people living in territories with the highest socioeconomic index)	€6469
Q2	€6540
Q3	€6811
Q4	€6678
Q5 (people living in territories with the lowest socioeconomic index)	€6845
Patients with insulin-treated diabetes	
Yes	€12,254
No	€5234
Concentration of reimbursements	
10% of patients with diabetes	More than €16,673 (51% of total reimbursements paid to patients with diabetes)
5% of patients with diabetes	More than €25,856 (35%)
1% of patients with diabetes	More than €59,748 (14%)

A very widely dispersed distribution of reimbursements paid to patients with diabetes was observed. Although the mean reimbursement was €6714/year, the median was only €2526 in 2012. 10% of patients with diabetes received more than €16,673 and 5% received more than €25,856. The concentration of reimbursements was therefore particularly high, with 10% of patients with diabetes (280,000 people) concentrating 51% of the €19 billion of reimbursements, 5% concentrating 35% of reimbursements and 1% concentrating 14%.

### Regression-based incremental approach: spending attributable to diabetes

Results from the GLM regression estimates are shown in Appendix 1. The fit of the model was assessed by using the goodness-of-fit Pearson’s Chi-square test, which was not statistically significant. The hypothesis of independence between the observed values and those estimated by the model assessing the fit of the selected model was then rejected.

The results of the fitted model were used to calculate the per-person spending attributable to diabetes (Table 4). The average spending attributable to diabetes clearly increased with age. For people 80 years and older, this expenditure was €6539 versus €3387 for the 50-59 age-group. Based on the average spending attributable to diabetes, the aggregate healthcare spending related to diabetes was €11.3 billion (all schemes).

**Table 4:** Estimated aggregate and mean economic burden of diabetes by age-group using the regression-based approach Source: CNAMTS\SNIIRAM 2012

	Regression-based approach	
	Total expenditure attributable to diabetes (95% CI)	Mean expenditure attributable to diabetes (95% CI)
Under 40 years	€203 million (€202–204 million)	€1644 (€1640–1648)
40–49 years	€462 million (€461–463 million)	€2312 (€2306–2318)
50–59 years	€1787 million (€1782–1791 million)	€3387 (€3379–3396)
60–69 years	€2802 million (€2795–2810 million)	€3271 (€3263–3280)
70–79 years	€3087 million (€3078–3096 million)	€4466 (€4454–4479)
Over 80 years	€3164 million (€3155–3173 million)	€6539 (€6520–6558)
All ages	€11,301 million (€11,072–11,332 million)	€3921 (€3910–3932)

### Incremental definition: matched-control approach

The additional expenditure measured by the matched-control approach corresponds to expenditure directly related to the treatment of diabetes, but also expenditure indirectly related to diabetes, for example expenditure related to obesity, a major risk factor for diabetes, or social deprivation, which can make the management of diabetes more complex and which is also linked to obesity and type 2 diabetes. According to this approach, the financial burden of diabetes was €7.7 billion (Table 5) with 58% due to outpatient care, 22% due to hospital care and 20% due to drugs.

**Table 5:** Breakdown of the €2.3 billion diabetes-specific expenditure Source: CNAMTS\SNIIRAM 2012

Method	Scope of health insurance reimbursements	Results
Method 1	Reimbursements within the population with diabetes	€19 billion
Method 2	Medicalized approach: reimbursements specific to diabetes	€2.3 billion
Method 3.1	Incremental definition: regression-based approach	€9.8 billion
Method 3.2	Incremental definition: matched-control approach	€7.7 billion
Method 4	Combination of medicalized and matched-control incremental approaches	€10 billion

### A global medicalized and incremental definition: diabetes-related reimbursed expenditure in France

#### Estimation of the reimbursed expenditure related to the management of diabetes

According to the medicalized approach, the total diabetes-specific reimbursed expenditure (see Table 1 for a list of diabetes-specific expenditure) was €2.3 billion in 2012 (Table 5). The excess reimbursements paid to patients with diabetes for all non-diabetes-specific expenditure represented €7.7 billion (Table 5). Diabetes-related reimbursed expenditure therefore represented a total of €10 billion (Table 5): 23% for diabetes-specific reimbursed expenditure and 77% for excess reimbursements due to diabetes. Diabetes-related reimbursed expenditure also represented 52% of all expenditure reimbursed to patients with diabetes (€19 billion). The per-patient cost of diabetes was €3387. Non-diabetes-related reimbursed expenditure (€9 billion, the difference between €19 billion, the global reimbursement received by people with diabetes and €10 billion the cost of diabetes among these €19 billion) corresponded to expenditure, which, in the absence of diabetes, would have theoretically been reimbursed to these patients, based on the expenditure of age- and gender-matched patients without diabetes.

**Fig. 2:**
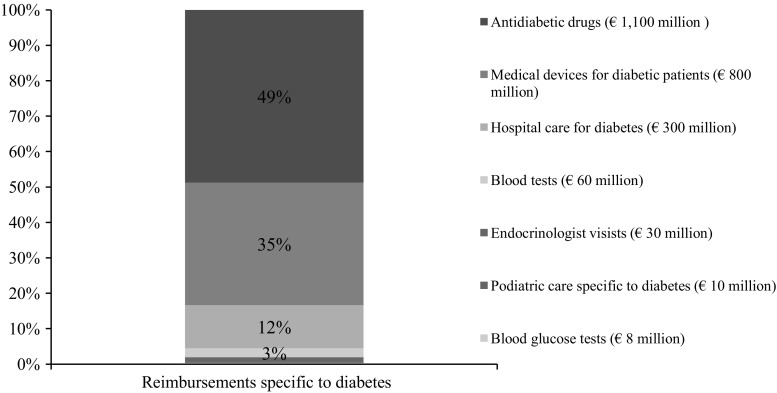
Breakdown of the €2.3 billion diabetes-specific expenditure Source: CNAMTS\SNIIRAM 2012

Antidiabetic drugs (oral hypoglycemic agents or insulin) represented an expenditure of about €1.1 billion in 2012, i.e., one-half (49%) of all diabetes-specific expenditure (€2.3 billion, see Fig. 2). Insulin therapy accounted for €400 million of this total €1.1 billion expenditure. Diabetes-specific medical devices (e.g., dip-sticks, insulin pens, insulin pump necessary materials) represented an expenditure of about €793 million, i.e., 35% of all diabetes-specific expenditure. Hospital stays specifically for diabetes represented a moderate share of diabetes-specific reimbursed expenditure (€270 million, i.e., 12% of diabetes-specific expenditure). Other types of expenditure, such as blood glucose and glycated hemoglobin assays, podiatrist fees, or endocrinologist visits (private practice and outpatient visits) represented a marginal share of diabetes-specific expenditure (4%).

### Burden of complications, nursing care, and sick leave

Cardiovascular diseases constitute a major complication or comorbidity of diabetes [26]. More than one quarter of patients with diabetes suffer from cardiovascular disease. The high prevalence of this disease in the population with diabetes as well as the more complex management due to the presence of comorbidities can explain a higher mean annual reimbursed expenditure for health care related to cardiovascular diseases for patients with diabetes compared to other patients. For example, for men over the age of 80 years, the mean reimbursed expenditure for drug treatments of hypertension was €114 for men without diabetes and €200 for men with diabetes (Table 6). Overall, by summing the excess reimbursements paid to the overall population with diabetes of all ages, the estimated diabetes-related reimbursed expenditure for antihypertensive drugs was €330 million, i.e., 20% of all reimbursements for these drugs (Table 7). Using the same methodology as for antihypertensive drugs, the diabetes-related excess reimbursed expenditure for lipid-lowering drugs was €240 million. Finally, the excess expenditure for all drugs used in the management of cardiovascular disease (antihypertensive, antiplatelet and lipid-lowering drugs, treatments for heart failure and peripheral artery disease) represented 7% of the financial burden of diabetes, i.e., €697 million.

**Table 6:** Mean reimbursements for patients with and without diabetes and excess reimbursements due to diabetes Source: CNAMTS\SNIIRAM 2012

Types of expenditure	Mean reimbursement for patients with diabetes	Mean reimbursement for patients without diabetes	Excess reimbursements due to diabetes
Medical fees			
General practitioner			
Men 80 years and older	€268	€195	€14 million
Women 80 years and older	€310	€223	€25 million
Drugs			
Antihypertensive drugs			
Men 80 years and older	€200	€114	€17 million
Women 80 years and older	€209	€111	€28 million
Lipid-lowering drugs			
Men 80 years and older	€122	€68	€11 million
Women 80 years old and over	€100	€46	€16 million
Auxiliaries			
Nurses			
Men 80 years and older	€1292	€453	€164 million
Women 80 years and older	€1938	€629	€377 million
Sick leave payments			
Men 50–59 years old	€1861	€916	€278 million
Women 50–59 years old	€1111	€690	€98 million
Hospital			
End-stage renal disease			
Men 80 years and older	€194	€87	€21 million
Women 80 years and older	€124	€32	€27 million
Ischemic heart disease			
Men 80 years and older	€172	€102	€14 million
Women 80 years and older	€93	€43	€14 million
Stroke			
Men 80 years and older	€106	€77	€6 million
Women 80 years and older	€94	€68	€8 million

**Table 7:** Breakdown of the non-diabetes-specific expenditure according to certain types of expenditure Source: CNAMTS\SNIIRAM 2012

Types of expenditure	Overall expenditure—for patients with diabetes	Excess reimbursements due to diabetes [percentage of all expenditure for patients with diabetes (according to the type of expenditure)]	Proportion of the excess reimbursement due to diabetes among the total reimbursed expenditure (overall population) (%)
Medical fees			
General practitioner	€627 million	€279 million (44%)	5.5
Cardiologist	€37 million	€16 million (43%)	9.4
Ophthalmologist	€27 million	€10 million (37%)	3.9
Nephrologist	€ 9 million	€6 million (67%)	21.3
Drugs			
Antiplatelet drugs	€131 million	€82 million (63%)	20.1
Antihypertensive drugs	€519 million	€330 million (64%)	19.4
Lipid-lowering drugs	€371 million	€240 million (65%)	20.3
Heart disease and peripheral artery disease	€101 million	€45 million (45%)	19.0
Lucentis^®^ (ranibizumab)	€72 million	€22 million (31%)	6.6
Medical devices			
Obstructive sleep apnea devices	€117 million	€82 million (70%)	22.5
Laboratory tests			
Cholesterol assays and renal function tests	€46 million	€28 million (61%)	13.4
Auxiliaries			
Nurses	€1865 million	€1425 million (76%)	30.3
Physiotherapists	€367 million	€99 million (27%)	3.5
Hospital			
Foot ulcer/amputation	€131 million	€112 million (85%)	44.8
End-stage renal disease	€362 million	€279 million (77%)	29.9
Chronic renal failure—acute renal failure	€171 million	€106 million (62%)	16.8
Ischemic heart disease	€317 million	€188 million (59%)	17.5
Heart failure	€196 million	€124 million (63%)	21.3

Another important diabetes-related complication, renal failure, was associated with high hospital stay expenditure. The diabetes-related excess reimbursements for hospital stays due to end-stage renal disease represented €279 million, i.e., 30% of all reimbursements paid for this disease to hospitals. The expenditure related to nephrologist visits attributed to diabetes (€6.3 million) represented 21% of all nephrologist visit expenditure. The last complication frequently associated with diabetes, diabetic foot ulcers and amputations, induced excess reimbursements of €112 million, i.e., almost one-half of all expenditure reimbursed for these diagnoses.

Nursing care expenditure presented a particularly high proportion of the expenditure due to the excess reimbursements to patients with diabetes that amounted to €1.4 billion, i.e., 30% of all reimbursed nursing care expenditure. For women with diabetes over the age of 80 years, the mean nursing care reimbursement was €1938 versus €629 for an age-matched woman without diabetes. Thus, in this age-group, the total reimbursed nursing care expenditure attributable to diabetes, i.e., induced by the excess reimbursements paid to women with diabetes of this age, was €377 million. The great majority of elderly patients treated with insulin, who are not always able to perform their injections by themselves, can partly explain this high use of nursing care in France.

Finally, diabetes and its complications can require intensive treatments that decrease the patient’s working capacity, leading to the payment of a cash allowance by national health insurance (daily sick leave allowances or disability pensions), in the smaller proportion of people in working-age groups. For example, a man with diabetes between the ages of 50 and 59 years received an average of €1861 of sick leave payments versus €916 (less than half) for an age-matched man without diabetes. The global excess payment of daily allowances to patients with diabetes represented a total of €528 million. 

## Discussion and conclusions

The four methodologies used in this study provided a range of different economic estimates of the burden of diabetes. Each method provides specific insight for policy makers to enhance diabetes management. Using a new, combined approach, diabetes-related reimbursed expenditure was estimated to be about €10 billion. We calculated that care for diabetes complications (cardiovascular diseases, chronic renal failure, diabetic foot ulcers, and amputations) and additional treatments accounted for the majority of the cost of diabetes care (€7.7 billion, 77%). Hospitalization for ischemic heart disease and heart failure accounted for €510 million. This result highlights the economic impact of cardiovascular risk prevention by monitoring HbA1c, lipids and blood pressure, but also by preventing smoking and obesity among patients with diabetes. Pay for performance programmes targeting general practitioners or disease management programmes for patients with diabetes could include these objectives in order to enhance follow-up of people with diabetes. These programmes may have a positive impact on the health status of patients with diabetes and, in the long term, should lower the overall health care expenditure by decreasing the number of events related to complications [27, 28].

Drugs (about €1.1 billion) represented one-half of the estimated cost of diabetes according to the medicalized approach. From a decision-maker’s point of view, this conclusion highlights the importance of promoting the most cost-effective drugs. The increasing variety of available pharmacological agents requires guidelines comprising therapeutic strategies that take these qualities into account. In France, the Haute Autorité de Santé (French Health Authority) released guidelines in 2013 recommending the use of metformin as first-line monotherapy. When dual therapy is required, the recommended first-line treatment is a combination of metformin and sulphonylurea. Insulin is the treatment of choice when oral therapy does not achieve the glycemic target. In 2015 the National Institute for Health and Care Excellence (NICE) published new guidelines, in which the costs of drugs were explicitly taken into account to choose the therapeutic strategy. These guidelines clearly state that if two drugs in the same class are appropriate, one should choose the option with the lowest acquisition cost. In line with the NICE guidelines, the Caisse Nationale d’Assurance Maladie des Travailleurs Salairiés (French National Health Insurance, CNAMTS), after consulting the French Health Authority, published comparisons of average treatment costs of various treatment strategies as well as comparisons of the price difference within each strategy between brand-name and generic drugs [29].

The high level of nursing care expenditure due to diabetes provides a different insight into the importance of developing new ways to provide care to insulin-treated patients, particularly elderly patients [30, 31], as, in the context of an ageing population and a high level of fee for services payment of nursing care, the growing number of patients on insulin will have a major impact on nursing care expenditure. Innovations promoting patient autonomy could be of particular interest. In this case, innovations may lead to productivity gains, contrary to the predictions of Baumol’s disease effect, which explains part of the increase of health care expenditure [32]. According to Baumol, productivity growth through innovation in the health care sector is often thought to be slower than in most other industries, partly because much of this expenditure concerns health care professional services. For this reason, the relative cost of health care tends to increase over time in relation to other consumer products—a phenomenon often referred to as the cost disease effect. A review of the payment system for nurses caring for insulin-treated patients in France could also be initiated. Firstly, bundled payment could replace fee for services payments of nurses when they provide long-term care for people with diabetes. Furthermore, National Health insurance could require evaluation of the rationale of a nurse’s intervention after a defined duration of treatment.

From a methodological perspective, the comprehensive approach provides an upper bound for the estimation of cost of illnesses. It provides an accurate picture of the overall expenditure of the population with a given disease. It also provides insight into the importance of top spenders: 1% of patients with diabetes accounted for 14% of the total expenditure of all patients. A particular focus on this population could help to curb the growth of health care expenditure for patients with diabetes.

The use of a medical and administrative database allows precise analysis of expenditure and identifies the types of expenditure providing the greatest contribution to the economic burden of diabetes. However, the limited number of sociodemographic variables may affect the results obtained by incremental approaches, as the estimated coefficient could be biased if variables highly correlated to diabetes are not available. The economic burden of diabetes could then be either underestimated or overestimated. For example, obesity is a strong risk factor for diabetes, and a low socio-economic level is associated with obesity and therefore with diabetes [16]. However, a low socioeconomic level may also be linked with other behaviors—smoking for example—or decreased or increased use of health care. Another example is that of genetic factors, which are also strong determinants of diabetes, and which display marked variability between ethnic groups. People belonging to certain specific ethnic groups may be more likely to develop diabetes, as well as other non-diabetes related diseases. They may also be derived from a lower socioeconomic background. To run a sensitivity test, we added to the control vector, surrogate variables a proxy of the individual’s financial situation and the ecological deprivation index [17] only available for metropolitan France after excluding the overseas territories for which it is not available. The economic burden of diabetes in metropolitan France was €10.7 billion when age and sex were introduced as the only control variables, but €10.3 billion when the ecological deprivation index was added. In the absence of control for the economic situation, the coefficient associated with diabetes was therefore probably overestimated. Other variables such as BMI, smoking, ethnicity, etc., were not available to be tested. Nevertheless, joint confounders may affect both the incidence of diabetes and the incidence of other diseases. The cost associated with diabetes could therefore be overestimated by not adjusting for these variables.

The matched-control approach, which compares the health care expenditure of subjects with and without the disease and attributes the differences to the cost of illness, requires the use of a reasonably comparable control group. Sensitivity analysis was conducted in order to test the impact of choosing 10-year age-groups instead of 5-year age-groups. No significant difference was observed, thereby confirming the robustness of our results. In a recent article about the cost of head and neck cancers in the United States [12], the matching variables used were age, sex, race, insurance status, the number of priority medical conditions (proxy for comorbidities) and year of data collection. We restricted the matching variables to age and sex, as race is not available in our database in which all individuals are insured by the national health insurance scheme. We did not add a proxy for comorbidities, as we considered age to be a good proxy to control for comorbidities for patients with diabetes [33]. A regional analysis of diabetes expenditure using the same methodology was also performed, but the results are not presented in this paper. Our results were compared with those based on the same database (Sniiram), but using a top-down approach [34]. In this study, based on the same population (French population covered by the health insurance general scheme), in 2012, €6.2 billion were attributed to direct management of diabetes and its complications except for cardiovascular complications, end-stage renal diseases or gestational diabetes, which were estimated separately. The results of this study were also broadly consistent with those of earlier studies [6, 35], although it is difficult to perform more detailed comparisons, particularly due to differences in time (1999 or 2007 cost data), but also differences in population definitions and data sources (survey and then extrapolation to the French population). It could also have been interesting to apply the new methodology, a prevalence-based top-down regression approach, developed for cost-of illness studies based on massive recently published data [36]. This method was not available at the time of our study, but it would also required preliminary adaptations and tests in order to assess, in particular, the feasibility for application on a database comprising information about 59 million individuals. This could be the subject of further investigations on cost-of-illness methods.

This study highlights robust methods that can be used to estimate the cost of diabetes. These methods provide policymakers with diverse and accurate information on the components of the cost of diabetes and therefore shed new light on the debate concerning the public policies to be implemented. In this context, the static approach (2012) to the financial burden of diabetes adopted in this study could be usefully completed by a dynamic approach taking into account the growth of expenditure in relation to the increasing prevalence of the disease and particularly the development of diabetic complications. By validating these various methods, this study demonstrates the value of using these methods for other chronic diseases in order to improve the management of chronic diseases.

## Electronic supplementary material

Below is the link to the electronic supplementary material.

Supplementary material 1 (PDF 95 kb)
