# Supplementary material for: The economic burden of diabetes to French national health insurance: a new cost-of-illness method based on a combined medicalized and incremental approach
Source: Eur J Health Econ. 2017 Feb 11;19(2):189–201. doi: 10.1007/s10198-017-0873-y (PMC5813074; doi:10.1007/s10198-017-0873-y)
Supplement: Supplementary file 1 — Supplementary material 1 (PDF 95 kb) [file 10198_2017_873_MOESM1_ESM.pdf]

Appendix 1: Estimated impact of diabetes on health care expenditure, a regression based approach

Model Information

Distribution	Gamma
Link Function	Log
Number of Observations Used	58737502

Criteria For Assessing Goodness Of Fit

Criterion	DF	Value	Value/DF
Deviance	59E6	163907561.07	2.7905
Scaled Deviance	59E6	75570613.497	1.2866
Pearson Chi-Square	59E6	1603485595.1	27.2992
Scaled Pearson X2	59E6	739297134.06	12.5865

Analysis Of Maximum Likelihood Parameter Estimates

Parameter	Estimate	Standard Error	Wald 95% Confidence Limits		Pr > ChiSq
Intercept	7.2931	0.0011	7.2910	7.2952	<.0001
10 to 19 years old	-0.1450	0.0007	-0.1465	-0.1435	<.0001
20 to 29 years old	0.5268	0.0007	0.5253	0.5282	<.0001
30 to 39 years old	0.9422	0.0008	0.9408	0.9437	<.0001
40 to 49 years old	1.0470	0.0007	1.0455	1.0484	<.0001
50 to 59 years old	1.4287	0.0008	1.4272	1.4301	<.0001
60 to 69 years old	1.3935	0.0008	1.3920	1.3951	<.0001
70 to 79 years old	1.7057	0.0009	1.7038	1.7075	<.0001
80 years old and older	2.0886	0.0010	2.0867	2.0906	<.0001
Less than 10 years old	0.0000	0.0000	0.0000	0.0000	.
Women	0.0149	0.0004	0.0141	0.0156	<.0001
Men	0.0000	0.0000	0.0000	0.0000	.
People without diabetes	-0.7932	0.0009	-0.7950	-0.7914	<.0001

People with diabetes	0.0000	0.0000	0.0000	0.0000	.

Note: The scale parameter was estimated by maximum likelihood.

Source: CNAMTS/SNIIRAM-2012
